# Clinical comparative study of standard channel percutaneous nephroscope combined with flexible ureteroscope and traditional standard channel combined with microchannel percutaneous nephrolithotomy in the treatment of multiple renal calculi without hydronephrosis

**DOI:** 10.12669/pjms.38.7.5526

**Published:** 2022

**Authors:** Yuanshan Guo, Lijun Yang, Xin Xu, Chao Li

**Affiliations:** 1 Yuanshan Guo, Department of Urology, Shijiazhuang People’s Hospital, NO.365 Jianhua Nan Road, Shijiazhuang 050010, Hebei, China; 2 Lijun Yang, Department of Urology, Shijiazhuang People’s Hospital, NO.365 Jianhua Nan Road, Shijiazhuang 050010, Hebei, China; 3 Xin Xu, Medical Service, Shijiazhuang People’s Hospital, NO.365 Jianhua Nan Road, Shijiazhuang 050010, Hebei, China; 4 Chao Li Department of Urology, Shijiazhuang People’s Hospital, NO.365 Jianhua Nan Road, Shijiazhuang 050010, Hebei, China

**Keywords:** standard channel, percutaneous nephroscope combined with flexible ureteroscope, multiple renal calculi without hydronephrosis, surgical treatment

## Abstract

**Objectives:**

To evaluate the clinical efficacy of standard channel percutaneous nephroscope combined with flexible ureteroscope and traditional standard channel combined with microchannel percutaneous nephrolithotomy in the treatment of multiple renal calculi without hydronephrosis.

**Methods:**

Eighty patients with multiple renal calculi without hydronephrosis treated in Shijiazhuang People’s Hospital from January 2020 to October 2021 were randomly divided into two groups: the experimental group and the control group, with 40 cases in each group. Patients in the experimental group were treated with standard channel percutaneous nephroscope combined with flexible ureteroscopy lithotripsy, while those in the control group were treated with standard channel combined with microchannel percutaneous nephrolithotomy. The differences in operative time, postoperative hospital stay, intraoperative blood loss, calculus clearance rate, and number of channels between the two groups were compared and analyzed. Moreover, the changes of renal function indexes such as serum creatinine, urea nitrogen, blood β 2-microglobulin and blood uric acid were compared and analyzed between the two groups postoperatively; Renal static imaging technology was utilized to compare and analyze the renal parenchymal injury of the two groups postoperatively. The incidence of surgical complications such as pain, fever, urine leakage at the incision, chest tightness and chest pain within 72h postoperatively was compared and analyzed between the two groups.

**Results:**

The operative time, postoperative hospital stay and intraoperative blood loss in the experimental group were significantly lower than those in the control group, with statistically significant differences (P=0.00). The number of percutaneous renal channels established in the experimental group was significantly superior to that of the control group (P=0.00); No statistically significant difference can be seen in the calculus clearance rate between the two groups (P=0.17); Postoperative TNF-a, CRP, IL-6 and other inflammatory factors in the experimental group were significantly lower than those in the control group (TNF-a, CRP, P=0.00; Il-6, P=0.01), and cortisol level in the experimental group was significantly lower than that in the control group, which was statistically significant (P=0.00). Postoperative renal static imaging showed that the degree of renal injury in the experimental group was lower than that in the control group (P=0.00). No statistically significant difference was observed in renal function indexes such as serum creatinine, urea nitrogen, blood β2-microglobulin and blood uric acid between the two groups (P>0.05).

**Conclusion:**

Standard channel percutaneous nephroscope combined with flexible ureteroscope is a safe and effective treatment regimen for the treatment of multiple renal calculi without hydronephrosis, boasting of numerous advantages such as reduced number of channels, less bleeding, short operative time, low kidney injury, low impact on internal environmental factors such as inflammation and stress in the patients, short postoperative hospital stay, and low incidence of complications.

## INTRODUCTION

Renal calculus, as a common disease in urology, is mainly caused by the occurrence of crystalline substances in urine under the action of factors such as diet, environment, infection and age. When the concentration of urine reaches a certain level, it tends to saturate, causing crystals to accumulate locally, and renal calculus is formed as a result.^1^ Renal calculus can generally be divided into two types: single and multiple. Single renal calculus is generally mild and can be treated with surgery relatively easily. In contrast, multiple renal calculus or staghorn calculus is usually caused by infection. Large and untreated staghorn calculi may injury the kidney and deteriorate its function and/or lead to life-threatening sepsis, which occurs and develops rapidly. Surgical complete removal of calculus is an important goal to eradicate infection, relieve obstruction, prevent recurrence and protect renal function.^2^ It remains a clinically recognized challenge in the treatment of complex renal calculus.

To protect the renal function of the patient’s remaining kidney as much as possible, renal calculus in the patient’s body should be completely removed as soon as possible. Percutaneous nephrolithotomy is the preferred treatment for this disease, with its main advantages of small trauma, high removal rate of lithotripsy and low incidence of postoperative complications, which is extensively applied in clinical practice.^3^ However, there are blind spots in the visual field in patients with large calculi such as multiple calculi. In order to increase the effect of calculus clearance, the surgeon often increases the swing range of the ureteroscope, which will cause great injury to the renal parenchyma, and even serious complications such as massive bleeding. For the purpose of alleviating injury, the main channel is currently used to establish the standard channel, and the auxiliary channel is used to establish the microchannel. Nevertheless, with the establishment of multiple channels, severe injury to renal parenchyma is still caused, and hydronephrosis increases the difficulty of puncture in an obscure way and the risk of bleeding.^4^

It is considered in the study by Ketsuwan et al.^5^ that renal bleeding that requires a blood transfusion is one of the most severe complications after percutaneous renal calculus surgery. Multivariate analysis showed that only multiple channel punctures were independent risk factors during PCNL (P=0.038). Flexible ureteroscopy technology is a novel technology developed in recent years, which can enter the kidney via the natural cavity of the human body for lithotripsy. Flexible ureteroscope is slender and flexible at the end, and can enter most renal calyces without obvious injury to renal parenchyma. There is no shear force injury to the renal parenchyma due to changes in direction or angle during rigid ureteroscope surgery, has been proved to have a preferable therapeutic effect on larger renal calculus.^6^ In this paper, standard channel percutaneous nephrolithotomy combined with flexible ureteroscope and traditional standard channel combined with microchannel percutaneous nephrolithotomy were compared in the treatment of multiple renal calculi without hydronephrosis, and the results showed that the former had obvious advantages.

## METHODS

A total of 80 patients with multiple renal calculi without hydronephrosis admitted to our hospital from January to October 2021 were selected and randomly divided into two groups: the experimental group and the control group, with 40 cases in each group. Patients in the experimental group were treated with standard channel percutaneous nephroscope combined with flexible ureteroscopy lithotripsy, while those in the control group were treated with standard channel combined with microchannel percutaneous nephrolithotomy. In the experimental group, 13 females and 27 males were enrolled, aged from 35 to 70 years, with an average of 54.45±9.07 years. In the control group, 18 females and 22 males were enrolled, aged from 37 to 68 years, with an average of 53.78±9.76 years. No significant difference was observed in the comparison of general data between the two groups (P>0.05), which was comparable between the groups (Table-I).

**Table I:** Comparative analysis of general data between he two groups (*X̅*±*S*) n=40.

Indexes	Experimental group	Control group	t/χ^2^	p
Age (years old)	54.45±9.07	53.78±9.76	0.32	0.75
Male (%)	27 (67.5%)	22 (55%)	1.32	0.25
Calculus site				
Left kidney (%)	16 (40%)	14 (35%)	0.21	0.64
Right kidney (%)	20 (50%)	19 (47.5%)	0.05	0.82
Bilateral (%)	4 (10%)	7 (17.5%)	0.95	0.33
Calculus volume (cm^2^)	3.96±0.88	4.02±1.24	0.25	0.81
Calculus location				
Renal pelvis (%)	12 (30%)	15 (37.5%)	0.50	0.48
Renal calyces (%)	6 (15%)	7 (17.5%)	0.09	0.76
Renal pelvis + Renal calyces (%)	22 (55%)	18 (45%)	0.80	0.37
Associated symptoms				
Pain (%)	11 (27.5%)	13 (32.5%)	0.24	0.63
Hematuria (%)	13 (32.5%)	10 (25%)	0.55	0.46
Urinary tract infection (%)	7 (17.5%)	6 (15%)	0.09	0.76
No symptoms (%)	9 (22.5%)	11 (27.5%)	0.27	0.61

p>0.05.

### Ethical approval

The study was approved by the Institutional Ethics Committee of Shijiazhuang People’s Hospital on January 20, 2020 (No.[2020]07), and written informed consent was obtained from all participants.

### Inclusion Criteria:

- Patients under the age of 70;
- Patients who met the diagnostic criteria for multiple kidney stones by preoperative imaging examination^7^;
- Patients without hydronephrosis;
- Patients with indications for surgery;
- Patients who volunteered to join the study and signed the consent form;
- Patients without obvious mental disorders and able to cooperate to complete the study.

### Exclusion criteria:

- Patients with coagulation insufficiency and cardiopulmonary dysfunction;
- Patients with a history of upper abdominal and renal surgery;
- Patients complicated with tuberculosis and pyonephrosis
- Patients with single renal calculus;
- Patients with pregnancy or lactation;
- Patients with severe urinary tract infection who cannot be cured within a short period of time by anti-inflammatory treatment;
- Patients with severe mental disorders and unable to complete the study;
- Patients with other severe underlying diseases that cannot be corrected and cannot tolerate surgery.

General anesthesia was used in both groups. First, the ureteroscope was inserted into the bladder through the urethra in the lithotomy position, and the ureteral catheter was inserted into the ureter along the ureteroscope. In case of bilateral simultaneous surgery, bilateral ureteral catheters were indwelled, and the ureteral catheters were connected to a pressurized flushing system to establish artificial hydronephrosis. Subsequently, the patients were changed to a prone position with their waist raised. Ultrasound scan of the target kidney was performed, and the puncture needle was inserted into the middle calyx of the target kidney under the guidance of B-mode ultrasound. When the needle core was withdrawn, urine was found to flow out, the special guide wire was inserted, the needle sheath was removed, the skin was cut with a sharp knife, and the needle passages were successively expanded with fascial expander along the guide wire to the F16 fascial expander. At the same time, a peel-Away thin skin sheath was inserted, F16 fascial dilator was pulled out, and then a metal dilator was inserted to expand the original fistula to F24. The F24 sheath was inserted and the ultrasonic gravel probe was inserted to break the visible calculus in the field of vision and absorb them synchronously.

For calculus outside the blind area of the standard channel visual field, the experimental group put a short flexible ureteroscope with a length of 30cm into the renal pelvis via the outer sheath and continued to search for calculus. The calculus in other parts was taken out with a calculus retrieval basket or moved to the standard channel field of vision to replace the nephrolithotomy and then aspirated. Ultrasound was used to scan the kidney and the ureteral catheter was removed after the effect of calculus clearance was satisfied. The F5 ureteral stent (DJ tube) was indwelled, the nephroscope was removed, and No. 15 metal expander was placed. Subsequently, the F24 sheath was removed and the F16 peel-away thin skin sheath was placed. Renal fistula was successfully placed along the thin skin sheath, and the thin skin sheath was removed. 3mL NS was injected into the water sac to remove the thin skin sheath and fix the renal fistula. In the control group, the calculi outside the blind area of the visual field of the first standard channel were selected for auxiliary microchannel lithotripsis, and the puncture point was selected again. The fascia expander was used to gradually expand to F16, and the peel-Away sheath was inserted. Then, the F16 fascial dilator was removed and the ureteroscope was inserted through the peel-Away sheath, followed by pneumatic balling or holmium laser lithotripsy. Finally, F5 double J tube was placed, F16 renal fistula was indwelled in standard channel, and urinary duct F8 was indwelled in auxiliary microchannel. Kidney ureter bladder was re-examined one day postoperatively, and renal fistula was removed for patients without residual calculus. Patients with small calculus residue underwent extracorporeal shock wave lithotripsy two weeks postoperatively.

### Comparative analysis of surgery-related indicators between the two groups

The differences in operative time, intraoperative blood loss, calculus clearance rate, number of channels, length of postoperative hospital stay and other indicators between the two groups were compared and analyzed.

Venous blood was taken on an empty stomach on the morning of the first postoperative day, respectively, and the changes of postoperative blood cortisol, CPR, IL-6, TNF-a and other inflammatory factors and stress factors were detected and compared between the two groups. 3) *Comparative analysis of renal function and renal parenchymal injury:* Fasting venous blood was taken from the two groups on the morning of the first postoperative day to detect renal function indicators such as serum creatinine, urea nitrogen, blood β 2-microglobulin, and blood uric acid, and the differences of the above indicators were compared between the two groups. Blood β2- microglobulin is a sensitive indicator of glomerular filtration function, and its elevation can be caused by various lesions related to glomerular filtration function. The renal static imaging technique was used to compare and analyze the renal parenchymal injury of the two groups of patients postoperatively. Method for judging the results of the radionuclide renal static imaging: two experienced nuclear medicine physicians read the radiographs together. The area of interest (ROI) technique was used to delineated the plane area of both kidneys and the area of sparse or defective areas, and the ratio of the injuryd area to the area of both kidneys was calculated. According to the renal injury scoring criteria in the literature^8^, the degree of renal injury was divided into five grades: Zero point means no injury, with an area ratio of 0%. One point means uncertain or mild injury, with an area ratio of < 5%. Two points indicate mild injury, with an area ratio of 5%-10%. Three points represent moderate injury, with an area ratio of 10%-30%. Four points indicate severe renal parenchymal injury, with an area ratio of>30%. 4) The incidence of surgical complications such as pain, fever, urine leakage at the incision, chest tightness and chest pain within 72h postoperatively was compared and analyzed between the two groups.

### Statistical Analysis

All the data in this study were statistically analyzed by SPSS 20.0 software, and the measurement data were expressed as (*X̅*±*S*). Two independent sample T tests were used for data analysis between the experimental group and the control group. Paired T test was used for data analysis between the two groups before and after treatment, and χ^2^ test was used for rate comparison. P<0.05 indicates a statistically significant difference.

## RESULTS

The comparative analysis of surgical data between the two groups is shown in Table-II. The operative time, postoperative hospital stay and intraoperative blood loss of the experimental group were significantly lower than those of the control group, with statistically significant differences (P=0.00). The number of percutaneous renal channels established in the experimental group was significantly superior to that of the control group (P=0.00). No statistically significant difference can be seen in the calculus clearance rate between the two groups (P=0.17).

**Table II:** Comparative analysis of surgical data between the two groups (*X̅*±*S*) n=40.

Group	Operative time (min)	Postoperative hospital stay (min)*	Bleeding volume (ml)*	Calculus clearance rate (%)	Number of channels (number)*
Experimental group	33.76±12.65	4.79±1.25	34.87±12.58	33(%)	1
Control group	51.38±10.73	6.21±2.02	65.76±13.21	37(%)	2.42±0.91
t/χ2	6.72	3.79	10.70	1.83	9.86
p	0.00	0.00	0.00	0.17	0.00

* p<0.05.

Postoperative renal static imaging indicated that the score of renal injury in the experimental group was 0.85±0.21, while that in the control group was 1.62±0.49, indicating that the renal Injury degree of the experimental group was lower than that of the control group (P=0.00). Moreover, no statistically significant difference can be seen in renal function indexes such as blood creatinine, blood urea nitrogen, blood β2-microglobulin, and blood uric acid between the two groups (P>0.05) (Table-III).

**Table III:** Comparative analysis of renal function and renal parenchymal injury in the two groups (*X̅*±*S*) n=40.

Indexes	Experimental group	Control group	t	p
Renal injury score (points)*	0.85±0.21	1.62±0.49	9.13	0.00
Cr (mmol/L)	75.83±21.76	74.92±22.07	0.18	0.85
BUN (mmol/L)	5.52±1.17	5.48±1.21	0.15	0.88
Blood β2-microglobulin (mg/L)	3.47±0.34	3.53±0.72	0.48	0.64
Blood uric acid (μmol/L)	321.67±23.75	318.73±25.78	0.53	0.60

***Note:***

* p<0.05.

Complications within 72h postoperatively in both groups were mild (Clavien I-II). No moderate or severe surgical complications occurred, and patients with urine extravasation recovered after prolonged renal fistula preservation. The comparison of complications between the two groups showed that in the experimental group, there were four cases of incision pain, two cases of fever, , two cases of urine extravasation, , two cases of chest distress and , two cases of chest pain, with a complication rate of 25% (10/40), while that in the control group was 52.5% (21/30). The incidence of complications in the experimental group was significantly lower than that in the control group, which was statistically significant (P=0.01) Table-IV.

**Table IV:** Comparative analysis of the complication rate between the two groups (*X̅*±*S*) n=30.

Indexes	Incision pain	Fever	Urine extravasation	Chest tightness	Chest pain	Total
Experimental group	4	2	2	1	1	10 (25%)
Control group	7	4	5	3	2	21 (52.5%)
χ^2^						6.37
p						0.01

p<0.05.

## DISCUSSION

Multiple renal calculi are commonly seen in clinical work of urology, especially in developing countries or economically underdeveloped areas. It is caused by a failure to seek medical attention in a timely manner, resulting in calculus that takes longer to grow, or calculus formed by infection, such as struvite. Calculi caused by infection are characterized by loose texture and fast growth, which can easily lead to repeated urinary tract infection and renal impairment^9^. Multivariate analysis by Ozgor et al.^10^ showed that multiple calculi are the main predictor of complications of percutaneous nephroscope surgery. Multiple calculi or large-volume calculi are often more likely derive small calculi, which are characterized by small size, smooth surface, round shape and easy to move into the ureter to lead to obstruction. Therefore, once multiple renal calculi or staghorn calculi are diagnosed, timely treatment is required.^11^

Percutaneous nephroscopy has always been the gold standard for the treatment of complex renal calculi such as multiple renal calculi and staghorn calculi.^12^ Percutaneous nephroscope surgery can be divided into four types according to channel diameter: large channel, standard channel, micro channel and ultra micro channel. Large channels have been replaced by standard channels due to serious renal parenchymal injury and obvious complications. Standard channel PCNL boasts the characteristics of short operative time, rapid lithotripsy and less trauma, and therefore has prominent advantages in the treatment of renal calculus.^13^

Kidney is complicated in its internal structure and has a large number of renal pelvis and calyces, resulting in a large blind field in rigid nephroscope during single-channel lithotripsis, and the treatment of calculi outside the blind area often requires the re-establishment of channels. Patients with multiple calculi or staghorn calculi have a very high calculi residual rate after single-channel surgery.^14^ Clinically, increasing the swing angle of the rigid ureteroscope or re-establishing the channel is the preferred method to increase the calculus clearance rate. Both of these methods increase the incidence of renal parenchyma tears, resulting in serious consequences such as bleeding. Meanwhile, the study results of Perepanova et al.^15^ suggested that excessive injury to renal parenchyma would increase the occurrence of postoperative systemic infection syndrome and urinary sepsis. It was considered in the study of Kalkanli et al.^16^ that complex calculi are more challenging than those confined to the renal pelvis, and lower red blood cell counts and higher infections after surgery are caused by a larger range of nephroscope swings during surgery. Despite being able to increase the calculus clearance rate, to a certain extent, via the establishment of multiple channels intraoperatively to compensate for the blind area of the main channel visual field, the injury to the kidneys can also be increased.

For patients suffering from multiple renal calculi without obvious hydronephrosis, the difficulty of the channel establishment is greatly increased compared with those with hydronephrosis^17^, and the multiple channel establishment is more complicated than the first one. With the progress of lithotripsy, perfusion extravasation into the perirenal area will gradually increase, which will reduce the clarity of the ultrasound image. Additionally, intrapelvic bleeding may also affect ultrasound images. Therefore, puncture becomes more difficult as the number of channels established increases. Moreover, multiple channels will increase the injury to the renal parenchyma and increase the risk of bleeding. When multiple channels are established, the chance of bleeding and intervention is higher than that of single channels.^18^

Flexible ureteroscope (FURS) combined with percutaneous nephroscope has certain advantages in the treatment of complex calculi. Standard channel percutaneous nephroscopy can be performed simultaneously during ultrasound or dual-catheter lithotripsy and calculus clearance. With the cooperation of flexible ureteroscope, large calculi can be moved to the field of vision of rigid ureteroscope, giving full play to various advantages of flexible ureteroscope, such as small ureteroscope size and flexible bending of the end, which can enter any renal calyces. In this way, the disadvantages of rigid ureteroscope can be compensated, and the drawbacks of re-establishing channels can be avoided. In combined surgery, the advantages of both flexible and rigid ureteroscopes can be brought into full play simultaneously. According to the study of Yanaral et al.^19^, the combination of FURS and microchannel percutaneous nephroscope is an effective choice for the treatment of multiple renal calculi of 10-30 mm, boasting the advantages of significantly lower complication rate, less surgical time, shorter learning curve and shorter hospital stay. Huang et al.^20^ reported postoperative fever (9.6%) is the most common complication of flexible ureteroscopy combined with microchannel lithotripsy, which may have a close bearing on poor water return during microchannel lithotripsy and low pressure in the renal pelvis. Anterograde surgery and retrograde surgery are the preferred methods of flexible ureteroscopy, the former being performed by entering the collecting system through the percutaneous renal channel, while the latter being performed by entering the collecting system through the ureter. Anterograde surgery combined with percutaneous nephrolithotomy (PCNL) is effective and safe in the treatment of renal calculus of 2-3cm size. It is suggested to weigh the advantages and disadvantages according to the individual characteristics of patients.^21^ The risk of flexible ureteroscope surgery is significantly lower than that of rigid ureteroscope. Anterograde surgery combined with percutaneous nephrolithotomy (PCNL) is effective and safe in the treatment of renal calculus of 2-3cm size. It is suggested to weigh the advantages and disadvantages according to the individual characteristics of patients.^21^ The risk of flexible ureteroscope surgery is significantly lower than that of rigid ureteroscope. Data from the study of Karagoz et al.^22^ showed that flexible ureteroscope has obvious advantages in terms of success rate, complication rate, length of hospital stay and infection rate, and can be used as an effective and safe alternative treatment for PNL. Renal model tests showed^23^ that reducing the pressure in the renal pelvis is the key to reducing postoperative complications. In this paper, a flexible ureteroscope was inserted into the standard channel, which resulted in a larger gap between the ureteroscope and the channel and a smoother backwater flow, thus resulting in a lower renal pelvis pressure. It was shown in the study of Hughes et al.^24^ that compared with rigid ureteroscope surgery, inflammatory markers such as cystatin C and CRP were significantly reduced after flexible ureteroscope surgery.

As shown in our study, standard channel percutaneous nephroscope combined with flexible ureteroscope is superior to traditional standard channel combined with microchannel percutaneous nephrolithotomy in the treatment of multiple renal calculus without hydronephrosis. The operative time, postoperative hospital stay and intraoperative blood loss in the experimental group were significantly lower than those in the control group, with statistically significant differences (P=0.00). The number of percutaneous renal channels established in the experimental group was significantly superior to that of the control group (P=0.00); No statistically significant difference can be seen in the stone clearance rate between the two groups (P=0.17); Postoperative TNF-a, CRP, IL-6 and other inflammatory factors in the experimental group were significantly lower than those in the control group (TNF-a, CRP, P =0.00; Il-6, P=0.01), and cortisol level in the experimental group was significantly lower than that in the control group, which was statistically significant (P=0.00). Postoperative renal static imaging showed that the degree of renal injury in the experimental group was lower than that in the control group (P=0.00).

### Limitations of this study

It includes a small sample size and there is no follow-up data, and the data of retrograde flexible ureteroscope have not been observed. In response to this, proactive countermeasures will be carried out to increase the number of cases, and further increase follow-up content and related data of other surgical methods, in order to objectively evaluate the benefits of the standard single-channel combination of flexible and rigid ureteroscopes for patients with multiple renal calculi without hydronephrosis.

## CONCLUSION

Standard channel percutaneous nephroscope combined with flexible ureteroscope is a safe and effective treatment regimen for the treatment of multiple renal calculi without hydronephrosis, boasting of numerous advantages such as reduced number of channels, less bleeding, short operative time, low kidney injury, low impact on internal environmental factors such as inflammation and stress in the patients, short postoperative hospital stay, and low incidence of complications.

### Authors’ Contributions:

**YG & CL:** designed this study, prepared this manuscript, are responsible and accountable for the accuracy and integrity of the work.

**LY:** Collected and analyzed clinical data.

**XX:** Data analysis, Significantly revised this manuscript.

## References

[1] Alshoabi SA, Alhamodi DS, Alhammadi MA, Alshamrani AF (2021). Etiology of Hydronephrosis in adults and children:Ultrasonographic Assessment in 233 patients. Pak J Med Sci.

[2] Ganpule AP, Naveen Kumar Reddy M, Sudharsan SB, Shah SB, Sabnis RB, Desai MR (2020). Multitract percutaneous nephrolithotomy in staghorn calculus. Asian J Urol.

[3] Large T, Assmus MA, Valadon C, Emmott A, Forbes CM, Agarwal D (2021). A Multi-institutional Review of Single-access Percutaneous Nephrolithotomy for Complex Staghorn Stones. Eur Urol Focus.

[4] Khadgi S, El-Nahas AR, Darrad M, Al-Terki A (2020). Safety and efficacy of a single middle calyx access (MCA) in mini-PCNL. Urolithiasis.

[5] Ketsuwan C, Pimpanit N, Phengsalae Y, Leenanupunth C, Kongchareonsombat W, Sangkum P (2020). Peri-Operative Factors Affecting Blood Transfusion Requirements During PCNL:A Retrospective Non-Randomized Study. Res Rep Urol.

[6] Takazawa R, Kitayama S, Tsujii T (2012). Successful outcome of flexible ureteroscopy with holmium laser lithotripsy for renal stones 2 cm or greater. Int J Urol.

[7] Reimer RP, Salem J, Merkt M, Sonnabend K, Lennartz S, Zopfs D (2020). Size and volume of kidney stones in computed tomography:Influence of acquisition techniques and image reconstruction parameters. Eur J Radiol.

[8] Mazaheri Tehrani M, Erfani M, Amirmozafari N, Nejadsattari T (2019). Evaluation of 99m Tc-MccJ25 peptide analog in mice bearing B16F10 melanoma tumor as a diagnostic radiotracer. Asia Ocean J Nucl Med Biol.

[9] McClinton S, Starr K, Thomas R, MacLennan G, Lam T, Hernandez R (2020). The clinical and cost effectiveness of surgical interventions for stones in the lower pole of the kidney:the percutaneous nephrolithotomy, flexible ureterorenoscopy and extracorporeal shockwave lithotripsy for lower pole kidney stones randomised controlled trial (PUrE RCT) protocol. Trials.

[10] Ozgor F, Yanaral F, Savun M, Ozdemir H, Caglar U, Sarilar O (2018). Comparison of miniaturized percutaneous nephrolithotomy and flexible ureterorenoscopy for moderate size renal stones in elderly patients. Kaohsiung J Med Sci.

[11] Pastore AL, Palleschi G, Silvestri L, Leto A, Ripoli A, Fuschi A (2016). Combined laparoscopic pyelolithotomy and endoscopic pyelolithotripsy for staghorn calculi:long-term follow-up results from a case series. Ther Adv Urol.

[12] Haider A, Mahmud SM (2022). Supracostal percutaneous nephrolithotomy, a safe and effective approach:A clinical audit. J Pak Med Assoc.

[13] Loftus CJ, Hinck B, Makovey I, Sivalingam S, Monga M (2018). Mini Versus Standard Percutaneous Nephrolithotomy:The Impact of Sheath Size on Intrarenal Pelvic Pressure and Infectious Complications in a Porcine Model. J Endourol.

[14] Falahatkar S, Moghaddam KG, Kazemnezhad E, Farzan A, Aval HB, Ghasemi A (2015). Factors affecting complications according to the modified Clavien classification in complete supine percutaneous nephrolithotomy. Can Urol Assoc J.

[15] Perepanova TS, Merinov DS, Kazachenko AV, Khazan PL, Malova YA (2020). [Prevention of infectious and inflammatory complications after percutaneous nephrolithotomy]. Urologiia.

[16] Kalkanli A, Cilesiz NC, Fikri O, Ozkan A, Gezmis CT, Aydin M (2020). Impact of Anterior Kidney Calyx Involvement of Complex Stones on Outcomes for Patients Undergoing Percutaneous Nephrolithotomy. Urol Int.

[17] Ng FC, Yam WL, Lim TYB, Teo JK, Ng KK, Lim SK (2017). Ultrasound-guided percutaneous nephrolithotomy:Advantages and limitations. Investig Clin Urol.

[18] de la Rosette J, Assimos D, Desai M, Gutierrez J, Lingeman J, Scarpa R (2011). The Clinical Research Office of the Endourological Society Percutaneous Nephrolithotomy Global Study:indications, complications, and outcomes in 5803 patients. J Endourol.

[19] Yanaral F, Ozgor F, Kucuktopcu O, Sarilar O, Ayranci A, Savun M (2019). Comparison of Flexible Ureterorenoscopy and Mini Percutaneous Nephrolithotomy in the Management of Multiple Renal Calculi in 10-30 mm Size. Urol.

[20] Huang JS, Xie J, Huang XJ, Yuan Q, Jiang HT, Xiao KF (2020). Flexible ureteroscopy and laser lithotripsy for renal stones 2 cm or greater:A single institutional experience. Medicine (Baltimore).

[21] Zewu Z, Cui Y, Feng Z, Yang L, Chen H (2019). Comparison of retrograde flexible ureteroscopy and percutaneous nephrolithotomy in treating intermediatesize renal stones (2-3cm):a meta-analysis and systematic review. Int Braz J Urol.

[22] Karagoz MA, Erihan IB, Doluoglu OG, Ugurlu C, Bagcıoglu M, Uslu M (2020). Efficacy and safety of fURS in stones larger than 20 mm:is it still the threshold?. Cent European J Urol.

[23] Doizi S, Uzan A, Keller EX, De Coninck V, Kamkoum H, Barghouthy Y (2021). Comparison of intrapelvic pressures during flexible ureteroscopy, mini-percutaneous nephrolithotomy, standard percutaneous nephrolithotomy, and endoscopic combined intrarenal surgery in a kidney model. World J Urol.

[24] Hughes SF, Moyes AJ, Lamb RM, Ella-Tongwiis P, Bell C, Moussa A (2020). The role of specific biomarkers, as predictors of post-operative complications following flexible ureterorenoscopy (FURS), for the treatment of kidney stones:a single-centre observational clinical pilot-study in 37 patients. BMC Urol.

